# Prenatal diagnosis of fetal midgut volvulus: a case report

**DOI:** 10.1186/s13256-022-03720-0

**Published:** 2022-12-28

**Authors:** Neelam Jain, Sandeep Singh Awal, Som Biswas, Tanushree Ghosh

**Affiliations:** 1 Jain Ultrasound, Jamshedpur, India; 2 Department of Radiology, Jeevandeep Diagnostics, Jamshedpur, India; 3 Le Bonheur Children’s Hospital, University of Tennessee Health Science Center, Memphis, USA; 4 Criticare Asia Hospital, Mumbai, India

**Keywords:** Fetal midgut volvulus, Malrotation, Ultrasound, Intrauterine, Whirlpool sign

## Abstract

**Background:**

Fetal midgut volvulus is an uncommon yet potentially life-threatening condition. Prenatal diagnosis may pose a challenge, due to the paucity of specific signs and symptoms. Timely prenatal diagnosis of this condition is imperative to prevent fetal mortality and morbidity.

**Case presentation:**

We present a rare case report of fetal midgut volvulus, malrotation, and intestinal obstruction at 32 weeks of gestation in a 31-year-old multigravida Indian patient who presented with decreased fetal movements. Fetal ultrasound revealed midgut volvulus with proximal bowel obstruction and polyhydramnios. The patient underwent emergency surgery, which revealed intestinal malrotation and confirmed the diagnosis of midgut volvulus. Untwisting of the volvulus was done followed by Ladd’s procedure. Follow-up postoperative ultrasound was unremarkable.

**Conclusions:**

Delay in the diagnosis of fetal midgut volvulus leads to poor fetal and maternal outcomes. Hence, it is vital for radiologists, sonologists, and obstetricians to be aware of this condition while performing fetal sonography. Prompt diagnosis and surgical intervention are vital to reduce the morbidity and mortality associated with this condition.

## Background

Midgut volvulus refers to the twisting of bowel loops around the mesenteric artery [1]. This leads to vascular congestion, poor venous return and eventual bowel obstruction, bowel infarction, perforation, or peritonitis [1–6]. It is more commonly seen postnatally than in the prenatal period [2, 3, 6, 7]. When encountered prenatally, the diagnosis of midgut volvulus is often challenging in the absence of specific signs and symptoms [7]. Timely diagnosis of this life-threatening condition is imperative to prevent fetal morbidity and mortality [3, 8].

We present and discuss the prenatal diagnosis of a rare case of fetal midgut volvulus with malrotation and intestinal obstruction.

## Case presentation

A healthy 31-year-old multigravida Indian female, gravida 4, abortions 2, para 1 was referred to our ultrasound clinic with complaints of reduced fetal movements. Her anomaly scan was previously conducted at our clinic and was unremarkable.

**Fig. 1:**
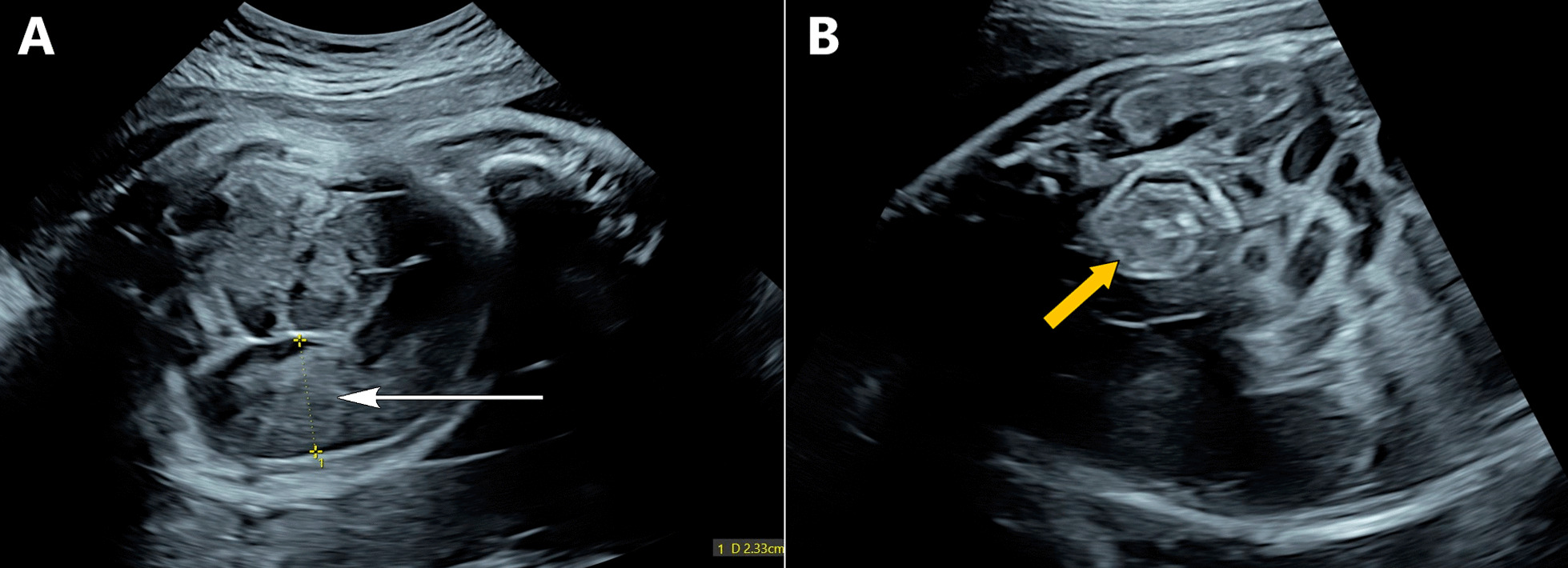
**A**,**B** Transverse transabdominal fetal sonography images demonstrating dilated bowel loops (white arrow) up to 2.3 mm (**A**) and classic “whirlpool sign” of twisted mesentery (yellow arrow) within the fetal abdomen (**B**)

The fetus was 33 weeks 6 days of gestation according to her last menstrual period (LMP). Ultrasound was performed using a GE Voluson E10 ultrasound machine with a C2-9 curved array probe, and the fetus’s gestational age was found to be 32 weeks and 3 days. Head biometry and femur length of the fetus were below the tenth percentile. Polyhydramnios was noted. The fetal abdomen appeared distended with significantly dilated bowel loops. The maximum diameter of dilated bowel loops was approximately 23 mm (Fig. 1A). In fetal mid-abdomen, echogenic bowel loops with the classic “whirlpool sign” were demonstrated (Fig. 1B). The perianal muscular sphincter complex appeared normal. No signs of bowel perforation were detected on fetal ultrasound.

The diagnosis of fetal volvulus with intestinal obstruction was made. The referring clinician was urgently informed about the findings, and the patient was referred to a tertiary care hospital. The patient was further counseled about the condition, regarding the need for emergency delivery and subsequent surgical intervention for the fetus.

Emergency lower-segment cesarean section (LSCS) was performed, and a female neonate weighing 1580 g was delivered. Apgar score was 9 and 10 at 1 and 5 minutes, respectively, and the vitals were within normal range.

**Fig. 2:**
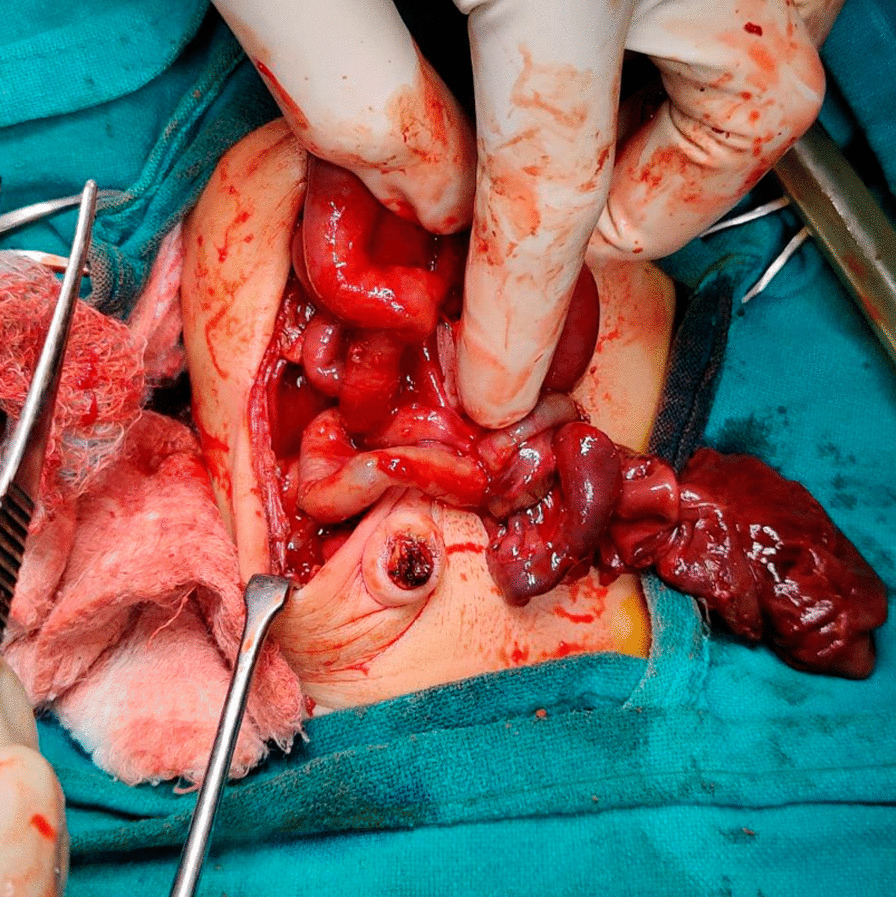
Intraoperative view of the intestine demonstrating midgut volvulus

On the basis of the fetal ultrasonography findings, emergency laparotomy was performed on the neonate at 4 hours after birth. Midgut volvulus was confirmed intraoperatively with the presence of malrotation (Fig. 2). No signs of bowel ischemia or necrosis were detected. Untwisting of the volvulus was performed followed by Ladd’s procedure. The postsurgical period was uneventful. Follow-up postoperative ultrasound done on day 1 postsurgery was unremarkable. On repeat visits, the infant’s growth was noted to be within the normal centiles for age. Follow-up ultrasound of the abdomen done at day 10 and at day 30 revealed nondilated bowel loops with normal peristalsis.

## Discussion

During the process of normal embryonic gut development, the fetal midgut undergoes a 270° counterclockwise rotation around the axis of the superior mesenteric artery between 4 and 8 weeks of gestation. Partial or complete failure of this rotation during embryogenesis leads to intestinal rotational abnormalities [9, 10]. Intestinal malrotation predisposes to midgut volvulus.

Fetal midgut volvulus is a rare entity diagnosed prenatally [3, 11]. It refers to the twisting of bowel loops around the mesenteric artery. This may lead to catastrophic complications, including intestinal obstruction, bowel ischemia, necrosis, perforation, and peritonitis [1].

Prompt diagnosis is vital to prevent undesirable maternal and fetal outcomes. Intrauterine diagnosis of fetal midgut volvulus is considered difficult and rare in literature [3, 9]. Ultrasound findings of fetal midgut volvulus include dilated intestinal loops and the classic “whirlpool sign” [12]. “Whirlpool sign” in midgut volvulus refers to the winding appearance of the mesentery and superior mesenteric vein wrapped around the superior mesenteric artery [12]. It has a high sensitivity, specificity, and accuracy for the detection of midgut volvulus [8]. Associated findings such as polyhydramnios, decreased fetal movements, and fetal ascites may be encountered [13, 14]. Fetal anemia may be seen in cases of intestinal volvulus. Increased peak systolic velocity (PSV) of the middle cerebral artery (MCA) on Doppler study can be indicative of fetal anemia, especially in the presence of fetal ascites, polyhydramnios, and dilated bowel loops [15]. Bartholmot *et al.* reported that decreased fetal movements in fetal volvulus was not an accidental finding. Moreover, the presence of a “fluid–fluid level” within the dilated bowel loops improves the diagnostic accuracy of findings on ultrasonography [16, 17]. The presence of a fluid–fluid level in dilated bowel loops indicates the absence of peristalsis and fetal intestinal distress [16].

“Coffee-bean sign” with or without whirlpool sign may be observed in fetuses with midgut volvulus [2, 16, 17]. It occurs in cases with closed-loop bowel obstruction and refers to the coffee-bean-like appearance of closely approximated dilated bowel loops [17, 18]

Detection of fetal midgut volvulus warrants an urgent neonatal surgical intervention. In the absence of associated anomalies, isolated midgut volvulus has a favorable prognosis postsurgery [14].

## Conclusions

Intrauterine midgut volvulus is an uncommonly reported yet life-threatening condition. Delay in the diagnosis of fetal midgut volvulus leads to poor fetal and maternal outcomes. Hence, it is pertinent for radiologists, sonologists, and obstetricians to be aware of this condition while performing fetal sonography. Prompt diagnosis and surgical intervention are vital to reduce the morbidity and mortality associated with this condition.

## Data Availability

The data and materials supporting the findings of this study are available on request from the corresponding author.

## Abbreviations

LMP: Last menstrual period
LSCS: Lower-segment cesarean section
PSV: Peak systolic velocity
MCA: Middle cerebral artery
